# The Taiji Model of Self

**DOI:** 10.3389/fpsyg.2019.01443

**Published:** 2019-06-21

**Authors:** Feng-Yan Wang, Zhen-Dong Wang, Rou-Jia Wang

**Affiliations:** ^1^Institute of Moral Education, Nanjing Normal University, Nanjing, China; ^2^School of Psychology, Nanjing Normal University, Nanjing, China; ^3^Nanjing Foreign Language School, Nanjing, China

**Keywords:** self, the Taiji Model of Self, self-structure, self-development process, person-making

## Abstract

An important theme in the development of self-psychology is the attempt by scholars to construct a self-model with universal cultural adaptability. Among them, representatives are the tripartite model of self-built by Triandis, the theory of the independent self and interdependent self-proposed by Markus and Kitayama, Yang Kuo-Shu's four-part theory of the Chinese self, Hwang Kwang-Kwo's Mandala model of self, and Shiah Yung-Jong's Non-self-Theory. However, these models have a difficult time explaining the structure and development of the Chinese self in Chinese cultural background. After pondering over Chinese traditional culture and the Chinese self, inspired by the archetype of Taiji diagram, in this paper, we construct the Taiji Model of Self. The Taiji Model of Self can not only properly represent the Chinese self-structure, but also explain the growth course of the Chinese self and four kinds of life realms of Chinese people with satisfactory cultural and ecological validity.

## Reflections on Self-Models in the Perspective of Chinese Culture

### Self and Ego

Since James introduced the concept of “self” into psychology in 1890, the “self” has been an important topic in the field. James divided a person's mental picture of the self into two categories: the “Me” as a separate object or individual a person refers to when describing their personal experiences, and the “I” as the self that knows who they are and what they have done in their life (James, 1890, p. 291–298, 329–337). This conception of the self was later followed by Cooley's (1902) looking-glass self-theory, Mead's (1934) social process theory of self, and Rogers' (1951) phenomenal field personality theory.

The psychodynamic personality structure constructed by Sigmund Freud, with the three layers of id, ego, and superego, as metaphorized by the “iceberg model,” has had a profound influence on personality psychology as well as social psychology. In the Freudian model, the ego (Latin for “I”), a person's sense of self, acts according to the reality principle, attempting to mediate between the impractical hedonism of the id and the equally impractical moralism of the super-ego; it is a set of psychic functions that is usually reflected most directly in a person's actions (Freud, 1923, p. 3–66). However, it might not match the Chinese tradition and reality, as the mainstream Chinese traditional culture accepts Mencius's theory of the original goodness of human nature and pays great attention to the discrepancy between humans and beasts. Therefore, in the ideology of most Chinese people, there is no place for id conception in the self-structure (Wang and Zheng, 2015, p. 571–573). Simultaneously, deeply influenced by Confucianism for more than two millennia, the Chinese are accustomed to the absence of the notion of God, in contrast to the monotheistic Abrahamic religions (Zhang, 2015). Thus, in Chinese culture, there is not a foundation for the existence of a superego analogous to God. To sum up, if the typical Western personality structure including the id, ego, and superego could be assimilated in a “sandwich,” we would say that the “pancake” is a typical Chinese personality model; that is, there is just a self but not an id and a superego in the Chinese personality.

Based on the criticism of Freud, Jung defined the “ego” as the center of the consciousness domain, but ego is unable to cover the subliminal psychological content of the wholeness of self. Jung called the transcendent center and wholeness of the psyche that embraces both conscious and unconscious the (big) Self. Therefore, in his view, the conscious ego is subordinate to the Self (Jung, 1951; Hwang, 2018a). The Jungian Self-absorbed the traditional Chinese thought from *The Book of Changes*, Taoism, and Buddhism that interlinks the self-concept we use in the present work (Jung, 1966). However, the relationship between Yin and Yang implied in the ego and Self has not been identified, and the ideology of Taiji has never been used to express the ontological wholeness of the Chinese Self.

### Cultural Self-Construal

With the development of cultural psychology in recent decades, constructing the self-model based on cultural context has become a hot topic (Yang and Lu, 2009; Markus and Kitayama, 2010; Hwang, 2011).

Markus and Kitayama (1991) put forward that people in different cultures had strikingly different constructions of the self and of others, so they divided the self into the interdependent self and independent self. They pointed out that most of the self-formed under the influence of Western culture is an independent self, showing as a bounded, self-contained, and autonomous entity, emphasizing the individual's separation from the social context. Conversely, the value system of East Asian culture is developed around the self-construal of interdependence, which is defined by social relations and emphasizes the connection with the surrounding context. In the amendment of their theory, Markus and Kitayama (2010) proposed that both types of self widely exist in each cultural environment and adjust according to the situation. There is not only distinction but also mutual production, organization, and promotion between the two types.

Moreover, based on cross-cultural comparisons, Triandis (1989) divided the self into the private self, public self, and collective self, three aspects with different probabilities of being expressed in different kinds of social environments. The private self involves a person's cognition of their own traits, states, or behaviors; the public self involves cognitions concerning the generalized other's view of the self; and the collective self involves cognitions concerning a view of the self that is found in some collective. Brewer and Gardner (1996), Kashima and Hardie (2000), and other scholars have since published similar tripartite self-theories. Sedikides and Brewer (2001) formalized these theories under the tripartite model of self. They argued that the self-concept consists of three fundamental self-representations: the individual self, in terms of unique personal traits; the relational self, in terms of dyadic relationships; and the collective self, in terms of group membership (Brewer and Gardner, 1996; Sedikides and Brewer, 2001).

The above theories have been supported by many empirical studies in recent years, but the interaction between culture and self in the theory reflects a lack of philosophical depth (Talhelm et al., 2014; Zhu and Ng, 2017, p. 85–96). Could these theories adequately explain the self-concept, self-structure, and self-development of Chinese people? It is doubtful.

### Theories of Chinese Self

Chinese anthropologist Fei Hsiao-Tung put forward that the modern Western society follows an “organization pattern,” while the Chinese society conforms to a “differential pattern;” that is, each Chinese person is constituted of a differential ordered web. Like the ripples formed by a stone thrown into a lake, each web has a self as its center, the circles extending out from the center to a certain distance according to the social relationship (Fei, 2008, p. 25–72). On this basis, Yang Yi-Yin used the division of “our own people” and “outsiders” to express the interpersonal relationship and self-structure of the present Chinese people. She provided a more detailed description of the “differential pattern” according to the five-degree interpersonal distance and the two dimensions of “ascribed relationship” vs. “interactive relationship” (Yang Y. Y., 2009).

In light of the interaction field of daily life, Yang Kwo-Shu divided the Chinese self into the “individual-oriented self,” “relationship-oriented self,” “family (group)-oriented self,” and “other-oriented self,” the first one being subjective and the latter three being objective. The conflicts between the self as subject and the secondary self as object are the main sources of the internal and interpersonal conflicts of Chinese people. Yang Kwo-Shu also admitted that his “four-part theory of Chinese self” is a further extension of the individual- and social-oriented theory (Yang and Lu, 2009, p. 86–129). On this basis, Lu Luo pointed out that under the influence of traditional Confucianism and modern Western culture, contemporary Chinese people have formed a “composite self” composed of an “individual-oriented self” and “socially oriented self,” which is a dialectical unity of “independence” and “interdependence” (Lu, 2003; Yang and Lu, 2009, p. 133–176).

Previous studies showed that the differences between the Eastern self and the Western self are mainly reflected in the relationship between the individual and the environment. The Western value system emphasizes control of the environment, the freedom of self, and the realization of individual potential, while the East Asian value system emphasizes the integration of the individual and environment, the restraint of self, and the consideration of the interests of the whole. Therefore, the Chinese self, as a typical case, is based on ethic relations and embedded in the social web (Yang and Lu, 2009, p. 2). Neuroscientific studies have shown that the medial prefrontal cortex (MPFC), a brain area related to self-concept, is activated in both self- and kinship-judgment conditions for Chinese subjects, while it is activated only in self-judgment condition for Western subjects (Zhu et al., 2007; Markus and Kitayama, 2010).

### The Mandala Model of Self and the Psychodynamic Model of Self-Nature

In recent years, the Mandala model of self, proposed by Hwang Kwang-Kwo, has produced an influence on the Chinese indigenous psychology. Hwang holds an ideal intention to construct a universal model to describe the well-functioning self in various cultures that may meet the principle of cultural psychology: “One mind, many mentalities.” Hence, it is different from the above substantial self-models of indigenous psychology in that it could be applied to any culture (Hwang, 2011, 2018a; Shiah, 2016). Inspired by the Borobudur Tower, it aims to illustrate the relationship between an individual's action and his/her cultural traditions. Hwang (2011) argues that the so-called “self” refers to an individual who has been socialized with the ability of reflexivity, whose life world can be represented by a structural model with a circle inside a square (Mandala). The self as a psychological concept in the circle is situated in the center of two bidirectional arrows: One end of the horizontal arrow points at “action” or “praxis”; the other end points at “knowledge” or “wisdom.” The top of the vertical arrow points at “person,” and the bottom points at “individual.” The arrangement of these concepts means one's self is being impacted by several forces from one's lifeworld (Hwang, 2011; Shiah and Hwang, 2019). Recently, Hwang further proposed the psychodynamic model of Self-nature by integrating the Mandala model with Jung's Self theory. He presented the “ogdoad” as a symbol of the psychodynamic model of Self-nature, which is composed of two pyramids/Borobudur Towers; the upper one represents one's course of life, while the opposite one represents one's collective unconscious. The ogdoad represents the topography of the conscious, personal unconscious, and collective unconscious, and the Mandala model of self is a cross-sectional slice of it, representing one's current self, existing at a particular moment of one's life; it can be conceptualized as a tridimensional model (Hwang, 2018a).

We admit that the Mandala model of self and the psychodynamic model of Self-nature have a good intention of achieving cultural compatibility and universality to facilitate the development of indigenous psychology. However, there are several issues worthy of discussion under the background of Chinese culture. First of all, the Mandala model is a part of the psychodynamic model of Self-nature, drawing the fundamental views of Freudian psychoanalysis and Jungian analytical psychology that embody a deep understanding on the personality. However, the ogdoad, composed by two pyramids with the consciousness on the top and unconsciousness underneath, essentially continued from the “iceberg model” as a sandwich, which could not fit to the characteristics of Chinese personality adequately, as we analyzed above (Hwang, 2018a). Although the ogdoad has deep cultural origins, with symbolic Mandala roots in Tibetan Buddhism and the Borobudur Tower being a stupa of Theravada Buddhism located in Indonesia, both of these are unfamiliar to the general Han Chinese population. They might be highly valued by Buddhists, but the majority of Chinese people have followed Confucianism for more than 2000 years since Emperor Wu of the Han dynasty adopted the policy of “forbidding all schools, venerating Confucianism only” and set Confucianism as the official ideology in 134 BC (Feng, 2011, p. 227–230; Hou, 2011). This has ascertained that the self-construal is closely related to the thinking mode. Hence, the Mandala symbol might not conform to the representative thinking mode of Chinese people, which is known as holistic thinking symbolized by Yin and Yang (Yang, 2006; Talhelm et al., 2014; Wang, 2018). Furthermore, it uses the structure model of a circle inside a square, not a square inside a circle, in the Mandala diagram, which is obviously different from the “round outside but square inside” (smooth on the surface, but firm at heart) lifestyle advocated by traditional Chinese culture (Hou, 2011). Therefore, the Mandala model as well as the psychodynamic model of Self-nature may be adequate self-models with cultural universality, but in consideration of the implicit interaction between the thinking mode and the self-construction in the Chinese cultural context, it is also worth trying to use the representative symbols of Chinese culture, including Yin and Yang, to construct an implicit Chinese self-model with cultural particularity.

Afterwards, Shiah Yung-Jong made extensions on the basis of the Mandala model of self. He developed the Confucian three-layered Mandala Model of self-cultivation (ordinary person, scholar, and king), the Buddhist three-layered Mandala Model of self-cultivation (non-Buddhist, Śrāvaka/Pratyeka Buddha, and Buddha), and an inward multilayer-stereo Mandala Model based on *The Book of Changes* (Shiah and Hwang, 2019). In addition, Shiah made the first attempt to propose the Non-Self-Theory based on Buddhist teachings, as well as three ways to execute the self-cultivation principle, namely, giving up desires, displaying compassion, and practicing meditation to seek Buddhist wisdom. The transition from the self-state to the non-self-state is a deeply transformative experience of eliminating the sense of self and its psychological structures and overcoming the illusion of the self, leading to authentic, durable happiness (Shiah, 2016; Hwang et al., 2017). In line with the Mandala Model of self, Shiah further developed the essence and substance of Buddhism in his theory. Although it involves the analysis of Confucian and Taoist self-cultivation, the starting point is still the ontology and epistemology of Buddhism.

### The Present Work

As reviewed above, there are two different orientations of the construction of self-models. Some psychologists would like to build self-models and self-theories that are adapted to one unique culture (Lu, 2003; Fei, 2008; Yang and Lu, 2009; Yang Y. Y., 2009); while other researchers wish to build a self-model with cultural compatibility and universality (Hwang, 2011, 2018a). Although numerous psychologists in both West and China have tried to establish a self-model with cultural adaptability, it is difficult to provide a perfect explanation of the Chinese self-structure in the Confucian cultural context.

In our view, the construction of self is closely related to the thinking mode, as the Chinese thinking mode has an intense cultural specificity (Yang, 2006; Talhelm et al., 2014; Wang, 2018), so we aimed to build a Chinese self-model with cultural particularity instead of university. There have been numerous philosophical schools in Chinese history, but Confucianism, Taoism, and Buddhism have been the most influential. Overall, among these three schools of thought, Confucianism has contributed most to the self-construal of Chinese people, as it has been the official and mainstream ideology of China for more than 2,000 years. The Confucian philosophy has the characteristic of introversion; the theory of disposition (心性之學, xin xing zhi xue) in particular can be regarded as pertaining to the study of self-psychology. Hence, the research of the Chinese self can be deemed as a key to opening the gate of mysterious Chinese culture.

The representative self-model in the Confucian cultural context should have the following two features. First, it should embody the particularity of the thinking mode of Confucianism originated from *The Book of Changes* (I Ching) in the pre-Qin period, which could be called Yin-Yang holistic thinking (Talhelm et al., 2014; Wang, 2018). Second, it should contain two main self-categories emphasized by Confucianism, that is, the small self and the large self (Yang, 2006; Wang and Zheng, 2015, p. 79–80).

In sum, using the pivotal Chinese cultural symbol, “the first diagram of China”—the Taiji diagram—as a prototype, based on the classic cosmic view and the thought on human nature of traditional Chinese Confucianism (Qian, 2011, p. 35–36), this paper constructs an implicit self-model with more explanatory power for the self-structure, self-development process, and realms of person-making of Chinese people under the influence of Confucian culture. And the meanings of using Taiji diagram as the prototype of self are: (1) indicating that the general self-structure of Chinese people likes a pancake but not an iceberg or sandwich as Freud put forward; (2) manifesting that the development and the transformation of Chinese self are autonomously dynamic and dispense with an outside driving object (like god); and (3) conforming to the representative Chinese Yin-Yang thinking mode.

## The Chinese Self-Centrism and Structure of the Taiji Model of Self

### Western Individualism and Chinese Self-Centrism

In the study of Western and Eastern self in cultural psychology, the Western self is often considered as a one-fold “self,” that is, an individual-oriented, independent self; on the contrary, the Chinese or even East Asian self under the influence of Confucianism is regarded as a relationship-oriented and interdependent self that contains others besides the individual (Zhu et al., 2007; Talhelm et al., 2014; Zhu and Ng, 2017, p. 45–64). As a matter of fact, the self-structure involved in culture is not so simple.

From the perspective of species subjectivity, the development process of the Chinese self is not only the development process of the Chinese individual moral self, but also the evolution of the self-concept in Chinese history. According to the glyph semantic analysis of the Chinese character “我” (*wo*, self) in the inscriptions on bones or tortoise shells of the Shang Dynasty, the origin of Chinese characters, it is not difficult to find that the self-concept of the Chinese in the pre-Qin period was limited to the meaning of one's own self and just about the individual, which is the most primitive meaning of self (Yang, 2006; Wang and Zheng, 2015, p. 75–58). Even though in the Spring and Autumn Period and the Warring States Period, the celebrated Yangism, represented by Yang Zhu, still strongly advocated individualism and stated that “If everyone does not harm a single hair, and if everyone does not benefit the world, the world will be well-governed of itself. Everyone should mind their own business, neither giving nor taking from others, and be content with what he has, and in that way, one will be happy and also contribute to the welfare of the world” (Yang, 2016, p. 242–243). This shows that the self-concepts in both the East and West are consistent in their historical origins (Wang, in press). However, the Chinese self-concept followed a different path away from the Western self-concept since the rise of Confucianism in the late Eastern Zhou Dynasty as well as its adoption as the official ideology in the Han Dynasty. In particular, the “Three Cardinal Guides and Five Constant Virtues” ideology based on the interpersonal ethical relationship laid the foundation for the moral pursuit of “growing the large self and restraining the small self” in Confucianism for more than 2,000 years (Hwang, 2018b). Consequently, the transformation of the self-concept marks the transition and shaping of Chinese culture (Wang and Zheng, 2015, p. 75–81; Wang, 2018).

From the perspective of entity, no matter in what kind of culture, various levels of self-structure, consisting of independent self-regions belonging to individual categories and interdependent self-regions belonging to different levels of relations in social categories, coexist within every single person. A specific self-structure is activated according to the situation (Yang and Lu, 2009, p. 87–110; Markus and Kitayama, 2010). In Chinese culture, the self-model shaped by Confucian tradition does not simply emphasize sociality and collectiveness. The real embodiment of self-structure is the differential mode of association and hierarchical spread represented by “推己及人, (tui ji ji ren),” which means extending oneself out to others. In this ideological tradition, individuation has not been ignored but has been put in the central position in the structure of self. Compared with Western “individualism,” this tradition is regarded as Chinese “Self-centrism” (Fei, 2008, p. 25–34).

### Dialectics vs. Yin-Yang

In recent years, the interpretation and discussion of Chinese “Self-centrism” has become increasingly abundant. Spencer-Rodgers et al. (2009) put forward the “dialectical self” view based on an empirical comparison between Chinese and American subjects, and revealed the contradictory, changeable, and holistic nature of the Chinese self-concept. However, the concept of “dialectic” used to define self is actually generated from Western essentialism and dualism, which is quite different from the non-essential characteristics of Chinese traditional philosophy (Yang, 2006; Zhu and Ng, 2017, p. 97–130). Yang Chung-Fang analyzed the difference between the Yin-Yang model and this dialectical model and then put forward the “Yin-Yang self-mode” on the basis of the “Yin-Yang thinking mode,” which preliminarily described the structure of Yin-Yang in the Chinese self. However, as her main purpose was to explore the Yin-Yang thinking mode, she did not further discuss the interaction and mutual transformation between the large self (da wo) and the small self (xiao wo) based on Yin-Yang theory, and did not touch the concept of “Taiji (太極),” which is the top-level concept of Yin-Yang and symbolizes the root of Chinese philosophy. It is also worth discussing some other points, such as regarding the relationship between the large self and the small self as a whole and part as well as equating the small self to the individual self (Yang, 2006; Wang and Zheng, 2015, p. 79–82). On the basis of these psychological theories or viewpoints, considering the self-view and the worldview in each culture are often on a continuous line, the “Taiji diagram” (diagram of the universe) in Chinese traditional cosmology could be taken as a key to unlock the mystery of the Chinese self.

**Figure 1:**
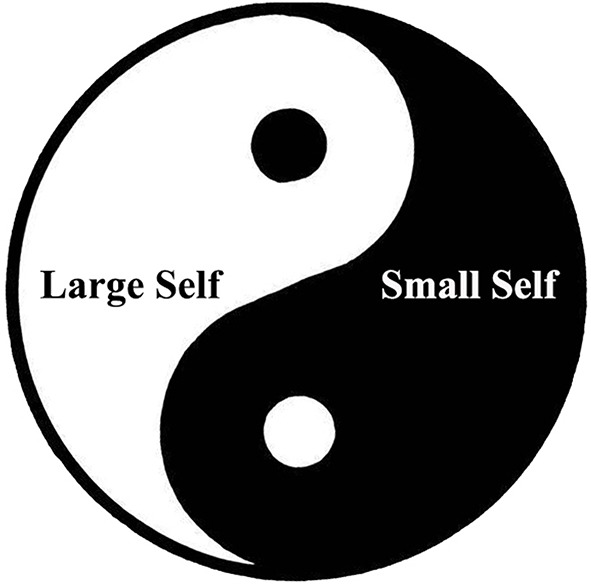
The Taiji model of self.

Taiji, originated from the cosmology in the I Ching, is the reflection of the relationship between Yin (陰) and Yang (陽) and is the embodiment of supreme noumenon Tao in Chinese metaphysics. The cosmic view in The Great Treatise of I Ching constructed the foundation of the whole Chinese traditional philosophy. As the treatise states, “The universe of Yin and Yang is called Tao”; “There are Changes in the Taiji, and they generate the two primary forces of Yin and Yang. The two primary forces generate the four images. The four images generate the eight trigrams.” The main parts of Taiji diagram are Yin and Yang, but it is totally different from dualism. The contrariety, coexistence, mutual dependence, and unity of Yin and Yang are the real principles implicated in the theory. The interpretation of the world and the universe based on Yin and Yang is both the ontology and epistemology of Chinese traditional philosophy. In addition, the Yin-Yang thinking mode, including the holistic thinking and dialectical thinking styles, is the most classic Chinese mode of thinking (Zhang and Cheng, 1991; Yang, 2006; Wang, 2018). Moreover, the Yin-Yang mode in Taiji diagram is intrinsically different from Hegel's dialectics inherited from Aristotle's either-or formal logic. First, the unity of opposites between Yin and Yang does not exist independently, like two aspects in a dialectical contradiction, but rather, both sides are inclusive of each other. Then, instead of achieving the resolution of contradictions through “sublation,” the aim of the mutual interaction between Yin and Yang is to attain an integration. The changes in this process are simply the expression of “Tao” (Li, 2013). In this way, after ruminating over the conception and structure of the Chinese self in the Chinese cultural context, inspired by the archetype of Taiji diagram, we constructed the “Taiji Model of Self” (Figure 1).

### Self-Structure in the Taiji Model of Self

#### Constitution and Contents

As shown in Figure 1, in the Taiji Model of Self, the whole circle on the outside representing “Taiji” refers to the whole “self.” Generally, one person has only one self, but it can be divided into a large self and a small self. According to the general metaphorical system, Yin (black part) usually signifies the nature of small, narrow, and dark, so it is used to denote the small self that represents the interests of the minority (ingroup); while Yang (white part) the nature of big, broad, and bright, so used to denote the large self that represents the interests of the majority (Yang, 2006; Wang and Zheng, 2015, p. 79–82; outgroup).

The black dot in the white implies that the seed of the small self is hidden in the large self, which represents the human desires (人慾, renyu) that trigger one to focus on the private interest to satisfy them; this could cause the shrinkage of the large self under certain circumstances. Meanwhile, the white dot in the black implies that the small self contains the seed of the large self, which represents the heavenly principles (天理, Tianli) that promote one to take other persons or other things into hear; this could advance the extension of the small self. As long as one has the ambition (志, zhi) and takes the initiative to cultivate in practice, it could advance the extension of the self. Thus, even within the large self generally concerns the public interests, there still exist private desires; conversely, if the small self is generally concerns the private interests, it still contains moral origins referring to Tao xin (道心) or conscience (良心, liang xin) (Qian, 2011, p. 66–73, p. 149–154; Wang, 2001). The mutual inclusion of Yin and Yang in Taiji diagram reflects the identical relationship between the large self and the small self in the self-structure. In a specific time and space, the “self” must be in a relative position between the “utter small self” and “utter large self.” “Utter small self” refers to the self that is only bounded by the individual body entity. It originates from the concept of self-reflexive address and is the minimum self-concept that cannot subdivide, so it is the utter small self. According to Confucianism, as the level of individual moral cultivation continues to improve, individuals would no longer set the boundaries between the self and others on the edge of the body entity. They must constantly expand their self-boundaries so as to accommodate others who have a special relationship with them, such as parents, children, and spouses, and fit these extensions into their self-concept (Yang C. F., 2009, p. 367–378).

Moreover, with the gradual deepening of individual moral cultivation, the self-concept can also be extended to many others who have no special relationship with oneself. It can even be expanded to everybody and everything in the universe, at which point it reaches the supreme realm in Confucianism—the self of the “unity of heaven and human (天人合一, tian ren he yi)” or the “identity with all creatures (民胞物與, min bao wu yu).” It is the “utter large self” because it is the maximum of self-concept that cannot be enlarge anymore. The process from the “utter small self,” passing through various progressive stages of small self and large self, to the “utter large self” is the ideal self-realization and person-making course in Confucianism. It can be regarded as an internal compatible expansion but not an external conquering expansion (Yang C. F., 2009, p. 367).

Hereon, the concepts of the “small self and large self” and “minority and majority” are relative. Through the modification and improvement of the “organization pattern” of Fei (2008) and the “circles of self” of Yang (2006); Yang C. F. (2009), we hold that, in Chinese traditional culture, the contents of the small self and the large self-change along with changes on the other side. The small self and the large self are like concentric circles, with the center being the individual self (utter small self). The first circle near the center signifies the self-representing the interests of one's nuclear family and close relatives; the second circle signifies the self-representing the interests of one's extended family and intimate friends; the third circle signifies the self-representing the interests of one's clan (a family of lineage, 家族, jia zu), good neighbors, ordinary friends, and so forth; the outermost circle signifies the self-representing the interests of the whole world, which equals the utter large self. Therefore, the self of someone in a certain state or a certain situation is relative and not absolute. If looking inward, it is the large self-relative to the inner circles; if looking outward, it is the small self-relative to the outer circles (Wang and Zheng, 2015, p. 79–114). For instance, when the small self represents the interests of the nuclear family (parents and kids), the large self, represents the interests of the extended family; when the small self represents the interests of fellow villagers (鄉人, xiang ren), the large self represents the interests of fellow countrymen (國人, guo ren). In a word, for Chinese people, the boundaries between “minority” (ingroup) and “majority” (outgroup) vary with changes in the self.

#### Changes and Dynamics

The Taiji Model of Self is not only a static presentation, but also a dynamic reflection. *The Great Treatise of I Ching* says, “it renews everything daily: this is its glorious power. As begetter of all begetting, it is called change.” Meanwhile, the *Su Wen (Plain Questions)* says, “Yin and Yang serve as the law of the heavens and the earth, the fundamental principle of all things, the parents of change.” Just like the Yin and Yang in the Taiji Model of Self, the small self and the large self are ceaselessly changing and transforming, providing driving forces for self-development, and the boundary between them is transparent and elastic (Yang C. F., 2009; Wang and Zheng, 2015, p. 79). As *Mencius, Jin Xin* says, “In dire straits one could only benefit his/her own self. Once in success, he/she should share his/her goodness with the whole world.” The presentations of self-shown by the same individual in different life states and real contexts are different.

In the Confucian culture, moral cultivation proceeds according to the clues of ethics, i.e., taking the “utter small self” as the starting point and the “utter large self” as the final destination. The guiding principle of life in the Confucian classic *The Great Learning* is that “if the personalities are cultivated, the families will be regulated; if the families are regulated, the states will be rightly governed; if the states are rightly governed, the whole world will be in peace and tranquil.” Hence, the work of cultivation advocated by Confucianism is reflected in the process of development and transformation from the small self to the large self. As Confucius said, “restrain oneself and regain propriety (克己復禮, ke ji fu li)” and “extend oneself out to others (推己及人, tui ji ji ren)”; in these ways, the self can find inward and outward directions in the self-centric flexible network. The key word in Chinese “Self-centrism” is “extend (推, tui)”—extending the self to the family, extending the family to the state, extending the state to the whole world. In this sense, the development of the self equals the extension of the self, and in this process, the balance state that is “the Doctrine of the Mean (中庸, zhongyong)” would be realized, which can be explained as “neither excessiveness nor insufficiency.” The optimal result of self-extension is achieving the “unity of heaven and human,” so that the self will hold the universe (Fei, 2008, p. 25–42; Wang and Zheng, 2015, p. 73–81).

## The Theory of Integrated Harmony of Self and Realms of Person-Making

### The Theory of Integrated Harmony of Self

Supposing that the Western self-development is “self-transcendence” from id and ego to superego, the Chinese self-development is equivalent to the pursuit of morality and the growth of spirit, which can be called the “integrated harmony of self.” The core principle of the “Theory of Integrated Harmony of Self” is as follows: Through the persistent and enduring cultivation of the mind, the individual will control his/her desires so that the small self will be restrained and the large self will become increasingly expanded; the channel from small self to large self is gradually linked, the whole self-advances continuously, and the integrated harmony of self will be developed when it ascends to a certain state (Hall and Ames, 1998, p. 23–42; Yang and Lu, 2009, p. 3–5).

The “person-making” in Chinese Confucianism is actually the continuous cultivation of the self. On the one hand, it is the improvement of one's comprehensive quality; on the other hand, it is the behavior of enhancing the relationship between the individual and the surroundings. Through this process, people make themselves participating members of the universe and live naturally and smoothly in their proper place. The final condition is the return to the human nature of goodness; understood from a psychological perspective, it is a process of self-construction.

The realm of person-making reflects the level of moral awakening of the self, which affects one's stable moral behavioral response in a specific situation (Yang, 2006). The Chinese self-cultivation process is carried out intentionally or unintentionally according to the theory of integrated harmony of self.

The Taiji Model of Self, like the psychodynamic model of Self-nature proposed by Hwang (2018a), contains the inspiration of Jung's individuation theory, although this influence is connotative. According to the individuation theory, the process of individualization refers to an integration process toward Self, which is the center of personality. It can be also translated as “coming to Selfhood” or “Self-realization” (Jung, 1966; Hwang, 2018a). Jung interpreted it as the process by which a person becomes a psychological “individual,” that is, a separate, indivisible unity or “whole” (Jung, 1951, p. 275). The basic feature of this process is that, with the purpose of the perfection and development of personality, individuation does not shut the self out from the world but accommodates the world into oneself (Jung, 1951; Hwang, 2018a). Therefore, it achieves a similar result to the integrated harmony process of self-implicated in the Taiji Model of Self. The process of confrontation, transformation, balance, and integration between the small self and the large self-represented by Yin and Yang reflects the integration of consciousness ego and unconsciousness, thus promoting the development of Self and achieving the wholeness of personality integrity. However, the greatest difference between the process of individualization and the integrated harmony process of self is the direction. The former, as its name suggests, is inclined toward the integration of the conscious realm and the unconscious realm inside a person, in the context of Western individualism, with the purpose of being completely independent, which is an individual internal process (Jung, 1966). Meanwhile, in the context of Chinese Self-centrism, the latter focuses on the integration of individuals and their surroundings and relationships, with the ultimate goal of the achievement of the “unity of heaven and human,” which is a process of interaction between the external and internal (Tang, 2005). Hence, it caused the different indication of Yin and Yang. According to Jung's individuation theory, comparatively speaking, the unconscious realm, as the submerged part of the “iceberg,” could be associated with “Yin”; the concept of “shadow” is such an expression (Jung, 1951). However, the conscious realm, with the center of ego, is the part above the “water” and expressed in the daily life; this could be associated with “Yang.” We can conclude that the “coordinate system” of Jung's individuation theory is different from the Taiji Model of Self because of the cultural context.

### Four Realms of Person-Making in Self-Development Process

**Figure 2:**
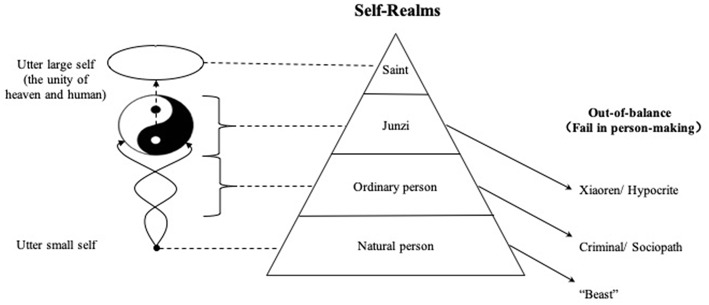
Schematic diagram of the integrated harmony process of self and four realms of person-making.

On account of the theory of integrated harmony of self, the Taiji Model of Self can be unfolded into a stereoscopic model, taking the process of self-development as the vertical axis. In other words, different realms of person-making could be divided according to the different levels of self-development. Chinese philosopher Feng You-Lan advanced the idea of four realms of life in his book *Xin Yuan Ren*: a natural realm, a materialistic realm, a moral realm, and a universal realm, based on the moral awareness and the dispositional development (Feng, 2014, p. 600–615). However, the division of the “materialistic realm” and “moral realm” is excessively simple; people in reality are often not purely materialistic or moral but rather a mixture of the two. According to the degree of the integrated harmony of self under the Confucian moral standard from bottom to top, the person-making realms reached through the cultivation of self can be divided into four categories: natural person (自然人, ziranren), ordinary person (常人, changren), junzi (君子, noble person, or gentleman), and saint (聖人, shengren) (see Figure 2).

#### Natural Person

If one behaves completely according to his/her nature, without any regard to the social ethics and legal system and turning his/her back upon others' interests and losses caused by his/her words and deeds in specific situations, then he/she is supposed to be a natural person. A natural person's words and deeds have nothing to do with good and evil. The person is totally ignorant; in this sense, he/she is not really socialized, so that he/she does not have the true sense of “human.” The states of infants or primitive men, such as eating when hungry, drinking when thirsty, and sleeping when tired, embodies the natural person. In the Taiji Model of Self, this realm corresponds to the “hundun (混沌, chaos)” field before the differentiation of Yin and Yang, or to the original point where the self is just germinating. At this point, the self is the utter small self, without any social connection (Feng, 2014, p. 601–602).

#### Ordinary Person

When one participates in social life, learning knowledge and conforming behavior norms that specifically refer to “five cardinal ethics” (wu lun, 五倫) mentioned by Mencius in the Pre-Qin period and “five cardinal virtues” (wu chang, 五常 or wu de, 五德) of humanity (仁, ren), justice (義, yi), propriety (禮, li), wisdom (智, zhi) and trust (信, xin) after Han dynasty through interactive activities, he/she will prove to be a member of the society. He/she understands and cares about the results of his/her words and deeds, and abides by social norms and laws. However, the wisdom and virtue of an ordinary person are average, and they may be flat throughout his/her whole life, whether in person-making or work. The normal state contains many subtypes, such as an honest person (moral person), mature person, and so on (Wang and Zheng, 2015, p. 528–575). An ordinary person is socialized in the real sense, and their words and deeds can be categorized as good and evil. According to the Taiji Model of Self, the Yin and Yang parts in the self-structure of ordinary people have been separated, and the transformation between them has been initiated. The large self has formed and presented a gradual extension trend, but the degree of its development is relatively elementary, far from achieving integrated harmony.

#### Junzi

If a person consciously abides by the code of conduct of a junzi (Wang and Zheng, 2015, p. 533–535) and is approved by the public, then he/she can be regarded as a junzi. In the Taiji Model of Self for a junzi, the extension of his/her self has reached a superior level that could accommodate a much larger scope of people compared with an ordinary person, not only him/herself and his/her close relatives but also his/her whole clan, neighbors, as well as compatriots. Hence, according to *The Great Learning*, a junzi could achieve self-cultivation (修身, xiu shen) and family harmony (齊家, qi jia), moving toward country management (治國, zhi guo), and eventually world peace (平天下, ping tian xia). A junzi is able to balance the relationship between the small self and the large self so as to coordinately facilitate the benefits of his/her self (small) and those of others in his/her large self. The selfish desire of a junzi should be restrained so that his/her social behavior is not affected by it; however, it still exists and remains to be cleared away. Otherwise, by force of circumstance, the self will shrink and give expression to the small self. The Chinese pursuit of the personality of a junzi has been likened to self-fulfillment under the orientation of Confucian collectivism (Yang and Lu, 2009, p. 120). A junzi has met superior moral standards, and the difference from the ordinary person can be determined by how much he or she makes profits for him/herself, others, and society. A junzi can generally achieve outstanding performance in his/her person-making or work, and his/her personality structure is relatively stable, which conforms to the standard of the “Doctrine of the Mean (zhongyong)” (Gong, 2006).

#### Saint

On the basis of a junzi, if a person has achieved a perfect moral character and superb wisdom, then he/she enters the realm of a saint. A saint can achieve extraordinary achievements, and his/her virtue has generally reached the supreme level, which realizes pure good in the unity of knowing and doing. He/she can attain the state of “following the heart's desire without overstepping the line,” as Confucius said in *The Analects*. In the Taiji Model of Self, the saint has reached the highest state of the complete integrated harmony of Yin and Yang, as well as the state of perfection with regard to the “unity of heaven and human” advocated by Confucianism since the Han dynasty (Feng, 2014, p. 693; Qian, 2011, p. 133–154).

The above four realms of person-making are just like the four stages of self-development, as well as four categories of self-representation. Each new stage will bring about qualitative changes in the mind and behavior. However, until the achievement of the saint realm, the development of the Chinese self and person-making is like “sailing against the current; not to advance is to fall back,” which means the process is not a one-way journey or once for all. If the self is influenced by inside desire or outside pressure in particular situation in a way that violates the moral cultivation, then the already-extended self will revert to the former state, leading to the failure and degradation of person-making. To be specific, if a person violates the basic ethics followed by a natural person, then he/she will be regarded as a “beast,” different from human; if he/she violates the laws that ordinary people must obey, he/she will degenerate into a “criminal” or a “sociopath”; if he/she violates the basic moral cultivation of a junzi, he/she will become a “xiaoren” (小人, despicable person, or villain) (see Figure 2).

As the mainstream ideology in most phases of Chinese history, Confucianism has had a profound influence on the Chinese conception of the self. The view of Yin and Yang in Confucian philosophy is derived from the “source of Chinese philosophy”—*The Book of Changes*. The description of the Chinese self-structure and the process of self-development in the Taiji Model of Self is consistent with the Confucian essence descended from the pre-Qin classical Confucianism (the orthodoxy of Confucius and Mencius, 孔孟之道) to the Song Ming Confucianism. We realize that Confucianism has experienced a process of secularization and aberrance in the secular living sphere during its development (Leung et al., 2002; Li, 2016). On the surface, secular Confucianism (xiaoren ru, 小人儒) ostensibly caters to the same self-view as classical Confucianism (junzi ru, 君子儒). However, under the influence of secular Confucianism, the false appearance of the “virtual self” will be produced; that is to say, it seems that the one's self is extending continually, but the “real self” can only be confined to the scope of the small self or even individual self. It intensely hinders to the development of self, causing the real self to be repressed so it cannot be extended and expanded. This has produced a very representative phenomenon in Chinese culture—that of the hypocrite (偽君子, wei junzi) who appears to be a junzi publicly but is a xiaoren privately (Wang and Zheng, 2015, p. 567–571). The “pollution” of the self-caused by the secularization and aberrance of Confucianism is actually a violation of the real Confucianism that takes “cautiousness in privacy” (慎獨, shen du) as a crucial discipline, so it should be classified into the category of the “failure of self-cultivation.” According to the Taiji Model of Self, it should be regarded as a phenomenon in which the transformation of Yin and Yang is blocked, so they are unable to integrate smoothly.

## Conclusions

First, although Chinese and Western psychologists are trying to build a self-model with cultural adaptability, the existing models have a difficult time adequately explaining the structure and development process of the Chinese self under the Confucian cultural background.

Second, taking the Taiji diagram as the prototype, the “Taiji Model of Self” could iconically represent the Chinese self-structure in the Confucian context, in which Taiji is the whole self and Yin and Yang are homologous to the small self and the large self, respectively. The change and transformation between Yin and Yang reflects the personality cultivation in the Confucian tradition of restraining the small self to extend the large self.

Third, the Chinese self-development process is carried out in accordance with “the theory of integrated harmony of self.” Four life realms can be distinguished according to the level of self-development, which are, from bottom to top, natural person, ordinary person, junzi, and saint. Falling to the state of a beast, xiaoren, criminal, or hypocrite indicates failure in self-cultivation and person-making.

## Author Contributions

F-YW constructing the theory and writing the paper. Z-DW writing the paper and revising the theory. R-JW revising the paper.

### Conflict of Interest Statement

The authors declare that the research was conducted in the absence of any commercial or financial relationships that could be construed as a potential conflict of interest.

## References

[B1] Brewer M. B. Gardner W. (1996). Who is this we? Levels of collective identity and self-representations. J. Pers. Soc. Psychol. 71, 83–93. 10.1037/0022-3514.71.1.83

[B2] Cooley C. H. (1902). Human Nature and the Social Order. New York, NY: Charles Scribner's Sons.

[B3] Fei H. T. (2008). From the Soil: The Foundations of Chinese Society. Beijing: People's Publishing House.

[B4] Feng Y. L. (2011). History of Chinese Philosophy. Shanghai: East China Normal University Press.

[B5] Feng Y. L. (2014). Six Books of Zhen Yuan. Beijing: Zhonghua Book Company.

[B6] Freud S. (1923). The ego and the id, in Standard Edition, Vol. 19, ed Strachey J. (London: Hogarth Press), 11–21.

[B7] Gong Q. (2006). The ideal of gentleman personality in ancient China. Stud. Ethics 1, 23–28. 10.15995/j.cnki.llxyj.2006.01.006

[B8] Hall D. L. Ames R. T. (1998). Thinking From the Han: Self, Truth, and Transcendence in Chinese and Western Culture. Albany, NY: State University of New York Press.

[B9] Hou H. H. (2011). Discussion the mandala method of the early esoteric Buddism. Stud. World Rel. 3, 30–41. 10.3969/j.issn.1000-4289.2011.03.004

[B10] Hwang K. K. (2011). The mandala model of self. Psychol. Stud. (Mysore). 56:329 10.1007/s12646-011-0110-1

[B11] Hwang K. K. (2018a). A psychodynamic model of self-nature. Counsel. Psychol. Quart. 10.1080/09515070.2018.1553147

[B12] Hwang K. K. (2018b). Five virtues: scientific approach for studying confucian ethics and morality. Int. J. Sci. Res. Methodol. 10, 176−198.

[B13] Hwang K. K. Shiah Y. J. Yit K. T. (2017). Eastern philosophies and psychology: Towards psychology of self-cultivation. Front. Psychol. 8:1083. 10.3389/fpsyg.2017.01083 28706498 PMC5489623

[B14] James W. (1890). The Principles of Psychology. New York, NY: Henry Holt and Company.

[B15] Jung C. G. (1951). The Collected Works of C. G. Jung, Vol. 9 (Part 2): Aion: Researches into the Phenomenology of the Self. Princeton, NJ: Princeton University Press.

[B16] Jung C. G. (1966). The relations between the ego and the unconscious, in Two Essays in Analytical Psychology Collected Works of C.G. Jung, Vol. 7, ed Hull R. F. C. (London: Routledge), 127–241.

[B17] Kashima E. S. Hardie E. A. (2000). The development and validation of the relational, individual, and collective self-aspects (ric) scale. Asian J. Soc. Psychol. 3, 19–48. 10.1111/j.1467-839x.2008.01271.x

[B18] Leung K. Koch P. T. Lu L. (2002). A dualistic model of harmony and its implications for conflict management in Asia. Asia Pac. J. Manag. 19, 201–220. 10.1037/a0037506

[B19] Li P. P. (2013). Indigenous research on Chinese management and Chinese traditional philosophies. Chin. J. Manag. 10, 1249–1261. 10.3969/j.issn.1672-884x.2013.09.001

[B20] Li X. (2016). The danger of Chinese exceptionalism. Manag. Org. Rev. 12, 815–816. 10.1017/mor.2016.41

[B21] Lu L. (2003). Defining the self-other relation: The emergence of a composite self. Ind. Psychol. Res. Chin. Soc. 20, 139–207. 10.6254/2003.20.139

[B22] Markus H. R. Kitayama S. (1991). Culture and the self: implications for cognition, emotion, and motivation. Psychol. Rev. 98, 223–253. 10.1037/0033-295x.98.2.224

[B23] Markus H. R. Kitayama S. (2010). Cultures and selves: a cycle of mutual constitution. Persp. Psychol. Sci. 5, 420–430. 10.1177/1745691610375557 26162188

[B24] Mead G. H. (1934). Mind, Self and Society. Chicago, IL: University of Chicago Press.

[B25] Qian M. (2011). An Outline of Neo-Confucianism in Song and Ming Dynasties. Beijing: Jiu Zhou Press.

[B26] Rogers C. (1951). Client-Centered Therapy: Its Current Practice, Implications and Theory. London: Constable.

[B27] Sedikides C. Brewer M. B. (2001). Individual self, relational self, and collective self: partners, opponents, orstrangers? in Individual Self, Relational Self, Collective Self, eds Sedikides C. Brewer M. B. (Philadelphia, PA: Psychology Press, 1–4.

[B28] Shiah Y. J. (2016). From self to nonself: the nonself theory. Front. Psychol. 7:124. 10.3389/fpsyg.2016.00124 26869984 PMC4740732

[B29] Shiah Y. J. Hwang K. K. (2019). Developing self-cultivation counseling psychology theories and empirical studies based on the Chinese cultural traditions of confucianism, Buddhism and Taoism: towards self-enlightenment psychotherapy. Chin. J. Guid. Counsel. 54, 1–20. 10.3966/172851862019010054001

[B30] Spencer-Rodgers J. Boucher H. C. Mori S. C. Wang L. Peng K. (2009). The dialectical self-concept: contradiction, change, and holism in East Asian cultures. Person. Soc. Psychol. Bull. 35, 29–44. 10.1177/0146167208325772 19106076 PMC2811254

[B31] Talhelm T. Zhang X. Oishi S. Shimin C. Duan D. Lan X.. (2014). Large-scale psychological differences within China explained by rice versus wheat agriculture. Science 344, 603–608. 10.1126/science.1246850 24812395

[B32] Tang Y. J. (2005). The Discussion on Unity of Heaven and Human. Hist. Chin Philos. 2, 5–10.

[B33] Triandis H. C. (1989). The self and social behavior in differing cultural contexts. Psychol. Rev. 96, 506–520. 10.1037//0033-295x.96.3.506

[B34] Wang F. Y. (2001). Argument about Li and desire in Confucian school of philosophy of the song and ming dynasties: a psychological review. Explor. Psychol. 21, 8–12. 10.3969/j.issn.1003-5184.2001.02.003

[B35] Wang F. Y. (2018). Questioning the Rice Theory: Also on the internal and external causes of Chinese preference for holistic thinking. Acta Psychol. Sinica 50, 572–582. 10.3724/SP.J.1041.2018.00572

[B36] Wang F. Y. (in press). Independent and interdependent self: Its emergence, transformation and formalization of Chinese self-construal from the evolution of culture and history. J. Nanjing Norm. Univ.

[B37] Wang F. Y. Zheng H. (2015). Chinese Cultural Psychology, 5th Edn. Guangzhou: Ji'nan University Press.

[B38] Yang B. J. (2016). Liezi Jishi (Collected Explanations on Liezi). Beijing: Chinese Publishing House.

[B39] Yang C. F. (2006). The Chinese conception of the self, in Indigenous and Cultural Psychology, eds Kim U. Yang K. S. Hwang K. K. (Boston, MA: Springer, 327–356.

[B40] Yang C. F. (2009). How to Understand Chinese People: A Symposium of Culture and Individuals. Chongqing: Chongqing University Press.

[B41] Yang K. S. Lu L. (2009). Chinese Self: Analysis With Psychology. Chongqing: Chongqing University Press.

[B42] Yang Y. Y. (2009). Guanxilization or categorization: psychological mechanisms contributing to the formation of the Chinese concept of us. Soc. Sci. China 30, 49–67. 10.1080/02529200902903800

[B43] Zhang D. N. Cheng C. Y. (1991). The Preferences of Chinese Thinking. Beijing: China Social Sciences Publishing House.

[B44] Zhang X. (2015). Construction of the faith of Chinese people. Theory Monthly 2015, 41–48. 10.14180/j.cnki.1004-0544.2015.12.008

[B45] Zhu Y. Ng X. H. (2017). Looking for the Chinese Self. Beijing: Beijing Normal University Press.

[B46] Zhu Y. Zhang L. Fan J. Han S. (2007). Neural basis of cultural influence on self-representation. NeuroImage 34, 1310–1316. 10.1016/j.neuroimage.2006.08.047 17134915

